# Value of the 14-gene molecular assay in efficacy assessment of neoadjuvant chemoimmunotherapy for non-small cell lung cancer

**DOI:** 10.3389/fmolb.2025.1619139

**Published:** 2025-07-10

**Authors:** Xuyan Lan, Xiaoyu Sun, Ruiqi Chen, Lihuan Zhu, Xiaojie Pan, Tianxing Guo

**Affiliations:** ^1^ Shengli Clinical Medical College, Fujian Medical University, Fuzhou, Fujian, China; ^2^ Department of Thoracic Surgery, Fujian Provincial Hospital, Fuzhou University Affiliated Provincial Hospital, Fuzhou, Fujian, China

**Keywords:** non-small cell lung cancer, 14-gene molecular assay, neoadjuvant therapy, pathological response, prognostic biomarker

## Abstract

**Objective:**

To evaluate the predictive accuracy of the 14-gene molecular assay in determining treatment response among patients with non-small cell lung cancer (NSCLC) undergoing neoadjuvant immunochemotherapy (nICT). Additionally, the study aims to investigate its correlation with tumor-infiltrating lymphocyte (TIL) levels and the status of tertiary lymphoid structures (TLS) in the tumor microenvironment.

**Methods:**

Patients with NSCLC who underwent nICT followed by surgical resection at Fuzhou University Affiliated Provincial Hospital between February 2019 and December 2022 were retrospectively included. Risk stratification was performed using the 14-gene quantitative PCR expression assay. The percentage of residual viable tumor cells (%RVT), TIL, and TLS within the primary lesion were evaluated through hematoxylin and eosin staining of surgical specimens. Subsequently, correlations were analyzed between the 14-gene molecular risk stratification and pathological response, as well as between the 14-gene molecular risk stratification and patient prognosis.

**Results:**

A total of 114 patients were included. The pathological complete response (pCR) rate was significantly higher in the 14-gene low-risk group, while the RVT was notably lower (both *P* < 0.05). Additionally, the low-risk group showed significantly elevated levels of TIL and positivity for TLS (both *P* < 0.05). Survival analysis revealed that patients in the low-risk group had markedly longer disease-free survival (DFS) compared to those in the intermediate-risk and high-risk groups (both P < 0.05). Univariate Cox regression analysis identified pathological TNM stage, vascular invasion, pathological response, and 14-gene molecular risk stratification as significant factors influencing DFS (all *P* < 0.05). Furthermore, multivariate analysis confirmed that the 14-gene risk stratification was an independent prognostic factor for DFS (HR = 2.496, *95% CI*: 1.264–4.931, *P* = 0.008).

**Conclusion:**

The 14-gene molecular assay demonstrated that low-risk status correlates with improved pathological response and prognosis, potentially attributable to higher TLS positivity rates and increased TIL infiltration. This assay offers critical insights for refining neoadjuvant treatment strategies in patients with NSCLC.

## 1 Background

Non-small cell lung cancer (NSCLC) is the most common type of lung cancer worldwide, comprising approximately 85% of all cases (Miao et al., 2024). Patients diagnosed at advanced stages typically face a poor prognosis (Chen et al., 2014). In recent years, immunotherapy, particularly immune checkpoint inhibitors targeting programmed death protein-1 (PD-1) and its ligand (PD-L1), has significantly improved outcomes for a subset of NSCLC patients (Kanabar et al., 2022). As an emerging therapeutic approach, neoadjuvant immunochemotherapy (nICT) involves the administration of chemotherapy and immunotherapeutic agents prior to surgical resection, which not only contributes to tumor downstaging but may also activate systemic anti-tumor immune responses, thereby enhancing long-term survival (Zheng et al., 2023). Nonetheless, substantial variability exists among individuals regarding their response to nICT, and reliable methodologies for predicting therapeutic outcomes remain scarce (Fei et al., 2023). To address this challenge, we aimed to identify appropriate predictive biomarkers to better assess treatment responses, optimize therapeutic strategies, and improve clinical outcomes for NSCLC patients undergoing nICT.

In recent years, the 14-gene molecular assay, a quantitative PCR-based method for molecular detection, has been widely utilized in prognostic studies for post-surgical NSCLC patients (Jiang et al., 2023). This assay assesses the expression levels of 11 cancer-related target genes (*BAG1, BRCA1, CDC6, CDK2AP1, ERBB3, FUT3, IL11, LCK, RND3, SH3BGR, Wnt3A*) and 3 reference genes (*ESD, TBP, YAP1*) within tissue samples to generate a continuous risk score. Using this score, patients are classified into low-risk, intermediate-risk, or high-risk categories. The reliability and clinical relevance of this molecular signature for risk stratification and prognostic prediction have been verified through multiple independent international validation cohorts (Jiang et al., 2023; Huang et al., 2024). Nonetheless, its utility in evaluating the efficacy of nICT in patients as well as its association with post neoadjuvant therapy changes in the immune microenvironment, including TIL levels and TLS positivity rates, remains to be clarified.

This study aimed to retrospectively analyze 14-gene molecular profiling in NSCLC patients who underwent surgical treatment following nICT. It also sought to evaluate the correlation between 14-gene risk stratification and pathological response rates after nICT, along with its association with the immune microenvironment, to explore potential biomarkers for predicting nICT efficacy in NSCLC and providing more precise guidance for individualized treatment of NSCLC patients.

## 2 Methods

### 2.1 Patients

Patients were collected from patients with NSCLC who underwent surgical resection following nICT at the Department of Thoracic Surgery, Fuzhou University Affiliated Provincial Hospital between January 2019 and December 2022. Eligibility criteria included: 1) receipt of nICT prior to surgery and 2) confirmation of an NSCLC diagnosis through both preoperative biopsy and postoperative pathological examination. Exclusion criteria included: 1) diagnosis of multiple primary lung cancers, 2) received prior targeted therapy, radiotherapy, or non-immunotherapy neoadjuvant treatments, 3) tissue samples of insufficient quality that failed to meet quality control standards for the 14-gene molecular assay. Clinical and pathological data were collected, including patient sex, age, smoking history, TNM staging, treatment cycles, pathological response, remission status, and follow-up outcomes.

### 2.2 14-Gene molecular assay

Formalin-fixed, paraffin-embedded tumor specimens from surgical resections were collected from all enrolled patients for 14-gene molecular quantitative PCR analysis (DetermaRx™, Burning Rock). This panel consists of 11 target genes (*BAG1, BRCA1, CDC6, CDK2AP1, ERBB3, FUT3, IL11, LCK, RND3, SH3BGR,* and *Wnt3A*) along with 3 reference genes (*ESD, TBP, and YAP1*). Expression data for the target genes were normalized against the reference genes, and a comprehensive risk score was calculated using an established mathematical algorithm. Based on these risk scores, patients were categorized into three groups: low-risk, intermediate-risk, and high-risk. The detailed procedures and methodologies for risk score calculation were conducted in accordance with previously published protocols (Kratz et al., 2013; Kratz et al., 2012).

### 2.3 RVT and histopathologic assessments of response

Using previously established methodology, we assessed the percentage of residual viable tumor (%RVT) in post-surgical specimens, which was determined by retrospectively analyzing H&E-stained tumor sections and calculating the ratio of the residual tumor area to the tumor bed area. The results from all examined sections were averaged to calculate the %RVT for each patient. Based on the %RVT, the pathological response was classified as either a pathological complete response (pCR) or a major pathologic response (mPR). Primary lesion pCR was defined as %RVT = 0%, while mPR was defined as %RVT ≤10% (Weissferdt et al., 2024).

### 2.4 Assessment of TLS and TIL

TLS and TIL were evaluated using H&E-stained sections of the primary lesion. TLS refers to organized, lymphoid node-like structures composed of lymphocytic aggregates containing a minimum of 50 immune cells (Vanhersecke et al., 2023). Tumors were classified as TLS-positive if at least one TLS structure was identified within the tumor, and TLS-negative if no such structures were observed. TIL, by contrast, refers to distinct clusters of lymphocytes that lack the structural organization or cellular composition of TLS (Cottrell et al., 2018). TIL was graded based on its proportion within the tumor: + (<10%), ++ (10%–50%), and +++ (>50%) (Hida et al., 2016).

### 2.5 Follow-up

The follow-up data for patients in this study were retrieved from the database of the Fuzhou University Affiliated Provincial Hospital. Overall survival (OS) was defined as the time span between the date of surgery and either the occurrence of death or the final follow-up. Disease-free survival (DFS) was defined as the period between the date of surgical resection and either the first recurrence or metastasis of the tumor, or the last follow-up. Patient follow-up was conducted through a combination of medical record reviews and telephone interviews, with the final follow-up concluded in January 2025.

### 2.6 Statistical analysis

Statistical analysis was conducted using SPSS 25.0 software. Continuous variables with a normal distribution were presented as mean ± standard deviation (x̄ ± s), and independent samples t-tests were used for comparisons between groups. For non-normally distributed continuous variables, the results were expressed as median (interquartile range) M (P25, P75), with comparisons between groups performed using the non-parametric Mann-Whitney U test. Categorical variables were represented as n (%), and group comparisons were conducted using either chi-square tests or Fisher’s exact tests, as appropriate. Correlations between 14-gene stratification and continuous variables were analyzed using Spearman’s rank correlation test, while correlations with categorical variables were assessed using Kendall’s tau-b test. Positive correlations were indicated by correlation coefficients *(r)* > 0, whereas *r* < 0 indicated negative correlations. Survival analysis was carried out using the Kaplan-Meier method, with survival curves generated and differences compared using the log-rank test. Univariate and multivariate Cox regression analyses were utilized to identify factors influencing DFS. Statistical significance was defined as *P* < 0.05.

## 3 Results

### 3.1 Clinical characteristics of patients

A total of 114 patients participated in this study, categorized into three risk groups: 50 patients (43.9%) in the low-risk group, 35 patients (30.7%) in the intermediate-risk group, and 29 patients (25.4%) in the high-risk group. Notably, the high-risk group exhibited a significantly higher proportion of clinical stage III/IV disease prior to treatment (*P* < 0.05, Table 1).

**TABLE 1:** Clinical characteristics of patients (n = 114).

Variables	Low-risk (n = 50)	Intermediate-risk (n = 35)	High-risk (n = 29)	*P*-value
Age (year), M (P25, P75)	60 (55, 66)	64 (61, 67)	62 (57,66)	0.178
Sex, n (%)				0.836
Female	12 (24.0)	8 (22.9)	9 (31.0)	
Male	38 (76.0)	27 (77.1)	20 (69.0)	
Smoking history, n (%)				0.772
No	30 (60.0)	24 (68.6)	18 (62.0)	
Yes	20 (40.0)	11 (31.4)	11 (38.0)	
Tumor diameter (cm), M (P25, P75)	2.0 (1.4,3.0)	2.5 (2.0,3.7)	3.0 (2.0,3.6)	0.172
Histologic subtype, n (%)				0.777
Adenocarcinoma	19 (38.0)	15 (42.9)	12 (41.4)	
Squamous cell carcinoma	31 (62.0)	19 (54.3)	16 (55.2)	
Others	0 (0.0)	1 (2.8)	1 (3.4)	
Pre-treatment clinical stage, n (%)				0.009
Stage I/II	30 (60.0)	18 (51.4)	8 (27.6)	
Stage III/IV	20 (40.0)	17 (48.6)	21 (72.4)	
Treatment cycles, n (%)				0.072
2	19 (38.0)	13 (37.1)	12 (41.4)	
3+	31 (62.0)	22 (62.9)	17 (58.6)	

### 3.2 Significant association between 14-gene risk stratification and pathological response

Significant correlations were identified between 14-gene risk stratification, pathological response, and %RVT. As risk stratification increased across low-, intermediate-, and high-risk groups, the proportion of non-mPR rose steadily, comprising 44% (22/50), 51% (18/35), and 79% (23/29), respectively. Conversely, the proportion of pCR declined, with rates of 16% (8/50), 29% (10/35), and 17% (5/29), respectively. These differences were statistically significant (*P* = 0.002, Table 2). Additionally, %RVT exhibited significant variation between the risk stratification groups (*P* = 0.031), with higher 14-gene risk stratification correlating with reduced pathological regression and elevated %RVT.

**TABLE 2:** Correlation between 14-gene molecular risk stratification and pathological response (n = 114).

Variables	Low-risk (n = 50)	Intermediate-risk (n = 35)	High-risk (n = 29)	*P*-value
Pathological response of primary lesion, n (%)				0.002
non-mPR	22 (44.0)	18 (51.4)	23 (79.3)	
mPR/non-pCR	20 (40.0)	7 (20.0)	1 (3.4)	
pCR	8 (16.0)	10 (28.6)	5 (17.2)	
%RVT, M(P25,P75)	10 (0,40)	10 (0,43)	45 (20,60)	0.031

non-mPR: non-major pathological response; mPR: major pathological response; pCR: pathological complete response; %RVT: percentage of residual viable tumor.

### 3.3 14-Gene molecular risk stratification as an independent prognostic factor for DFS

**FIGURE 1:**
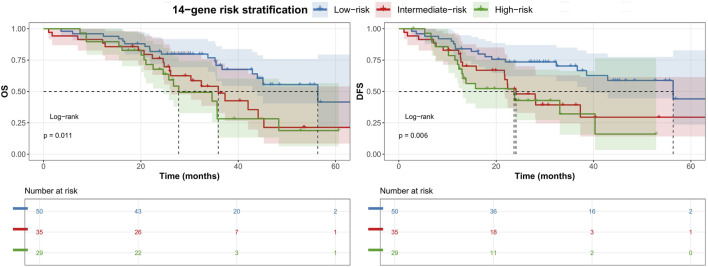
Significant differences in OS (left) and DFS (right) among 14-gene defined risk groups.

Univariate Cox regression analysis identified post-treatment TNM stage, lymphovascular invasion, pathological response, and gene risk stratification as prognostic factors for DFS in NSCLC patients undergoing nICT (Table 3). An elevated TNM stage was strongly associated with an increased risk of disease progression (*P* < 0.001), whereas pCR significantly correlated with prolonged DFS (HR = 0.237, *95%CI*: 0.107–0.526, *P* < 0.001). Further multivariate analysis confirmed that 14-gene molecular risk stratification (high-risk: HR = 2.496, *95%CI*: 1.264–4.931, *P* = 0.031) and pCR (HR = 0.135, *95%CI*: 0.027–0.671, *P* = 0.014) were independent predictors of DFS. Kaplan-Meier survival analysis demonstrated notable differences in OS and PFS among the 14-gene molecular risk stratification groups (Figure 1). Patients in the low-risk group had the longest median survival times, with significantly higher survival rates compared to the intermediate-risk and high-risk groups (*P* < 0.05).

**TABLE 3:** Univariate and multivariate Cox regression analysis of factors affecting patient DFS.

Variables	Univariate analysis		Multivariate analysis	
	*HR (95%CI)*	*P*-value	*HR (95%CI)*	*P*-value
Age	1.017 (0.985∼1.105)	0.307		
Sex (Female/Male)	0.989 (0.472∼2.074)	0.976		
Smoking history (No/Yes)	1.477 (0.904∼2.412)	0.119		
Treatment cycles (2/>2)	1.152 (0.975∼1.9667)	0.604		
Post-treatment TNM stage		<0.001		0.102
0	1		1	
1	1.642 (0.751∼3.587)	0.214	0.529 (0.136∼2.063)	0.359
2	3.293 (1.457∼7.439)	0.004	0.389 (0.094∼1.619)	0.194
3+	4.122 (1.956∼8.689)	<0.001	0.266 (0.067∼1.060)	0.060
Vascular invasion (No/Yes)	1.986 (1.112∼3.548)	0.020	1.164 (0.614∼2.206)	0.643
Pleural invasion (No/Yes)	1.736 (0.925∼3.257)	0.086		
Pathological response of primary lesion		0.001		0.050
non-mPR	1		1	
MPR/non-pCR	0.563 (0.291∼1.086)	0.086	0.626 (0.289∼1.356)	0.235
pCR	0.237 (0.107∼0.526)	<0.001	0.135 (0.027∼0.671)	0.014
Gene risk stratification		<0.001		0.031
Low-risk	1		1	
Intermediate-risk	2.522 (1.345∼4.728)	0.004	1.716 (0.877∼3.358)	0.115
High-risk	4.017 (2.171∼7.431)	<0.001	2.496 (1.264∼4.931)	0.008

DFS: Disease-free survival; HR: hazard ratio; CI: confidence interval; non-mPR: non-major pathological response; MPR: major pathological response; pCR: pathological complete response; %RVT: percentage of residual viable tumor.

### 3.4 Correlation analysis between 14-gene signature and pathological characteristics

The association between 14-gene molecular risk stratification and the pathological characteristics of the immune microenvironment was further analyzed and assessed. The analysis revealed a notable negative correlation between 14-gene molecular risk stratification and both TIL and TLS status (*P* < 0.05, Table 4). As gene risk stratification shifted from low-risk to high-risk, the incidence of high TIL infiltration (3+) declined, whereas the prevalence of low TIL infiltration (1+) increased.

**TABLE 4:** Correlation between 14-gene risk stratification and TIL and TLS.

Variables	Low-risk (n = 50)	Intermediate-risk (n = 35)	High-risk (n = 29)	*P*-value
TIL, n (%)				0.016
1+	12 (24.0)	16 (45.7)	16 (55.2)	
2+	23 (46.0)	15 (42.9)	11 (37.9)	
3+	15 (30.0)	4 (11.4)	2 (6.9)	
TLS, n (%)				0.020
Negative	8 (16.0)	11 (31.4)	13 (44.8)	
Positive	42 (84.0)	24 (68.6)	16 (55.2)	

TIL: tumor-infiltrating lymphocytes; TLS: tertiary lymphoid structures.

The proportion of TLS-positive cases decreased progressively with increasing gene risk stratification. Specifically, TLS positivity was identified in 42 (84.0%) patients in the low-risk group, 24 (68.6%) patients in the intermediate-risk group, and 16 (55.2%) patients in the high-risk group, with statistically significant differences observed between groups (P = 0.020). These findings revealed a notable trend: patients classified under higher 14-gene risk stratification exhibited reduced TIL infiltration and lower TLS positivity. This suggests that gene risk stratification may serve as a valuable tool in evaluating the status of the immune microenvironment.

## 4 Discussion

In this study, we conducted a 14-gene molecular assay on NSCLC patients undergoing nICT, alongside an evaluation of tumor microenvironment factors such as TIL and TLS, and demonstrated that the 14-gene molecular assay holds significant predictive value for pathological response and DFS. Specifically, patients classified into the high-risk group based on the 14-gene signature exhibited substantially lower pCR rates, markedly elevated %RVT, and significantly shortened DFS compared to those in the low-risk group. Furthermore, high-risk patients showed notably reduced proportions of TLS positivity and lower levels of TIL infiltration, indicating that the 14-gene molecular assay not only correlates with pathological response but also reflects the immune status of the tumor microenvironment. These findings provide important evidence for optimizing neoadjuvant treatment strategies in NSCLC patients.

As the 14-gene molecular risk stratification progresses from low to high risk, pathological response steadily decreases, characterized by significant reductions in the pCR rate and pronounced elevations in RVT. Cox regression analysis further confirms the independent prognostic value of the 14-gene molecular assay in predicting DFS, with high-risk patients exhibiting substantially lower DFS compared to those in the low- and intermediate-risk groups. Previous studies have established a strong association between the 14-gene molecular stratification and the pathological features of lung cancer, malignant biological behavior, and immune regulation, underscoring its ability to reflect tumor biology and patient prognosis (Ding et al., 2025). Among the 14 genes, BRCA1 is associated with DNA damage repair, and its low expression may enhance tumor cell sensitivity to treatment (Yoshida and Miki, 2004); conversely, high *Wnt3A* expression may promote tumor cell proliferation and differentiation, correlating with poorer pathological response and prognosis (Lin and Liu, 2021). Furthermore, elevated *CDC6* expression may promote tumor cell proliferation by regulating the cell cycle (Borlado and Méndez, 2008), while high *LCK* expression may enhance T cell activity, thereby improving immunotherapy efficacy (Moogk et al., 2016). These findings further highlight the promising application of the 14-gene molecular assay for evaluating nICT efficacy in NSCLC patients.

The 14-gene molecular assay was strongly correlated with immune cell infiltration within the tumor microenvironment. As the 14-gene risk stratification increased, patients showed markedly decreased levels of TIL infiltration and TLS positivity rates. The low-risk group exhibited significantly higher proportions of robust TIL infiltration (3+) compared to the high-risk group, along with notably elevated TLS positivity rates. These observations suggest that the 14-gene molecular test effectively reflects the immune status of the tumor microenvironment, underscoring its potential utility in assessing the efficacy of immunotherapy. Prior research has established the pivotal roles of TIL and TLS in shaping the tumor immune microenvironment, with high levels of TIL infiltration generally linked to stronger anti-tumor immune responses and improved prognoses (Noël et al., 2021; Ruffin et al., 2021). Similarly, the presence and maturity of TLS have been shown to enhance both local and systemic anti-tumor immune responses by facilitating the activation of B cells and T cells (Fridman et al., 2023; Teillaud et al., 2024). Genes within the 14-gene signature may play critical roles in immune microenvironment regulation. For instance, elevated *IL11* expression may suppress anti-tumor immune activity by fostering the development of an immunosuppressive microenvironment (Tang et al., 2024), while increased *ERBB3* expression may impact immunotherapy outcomes by modulating the interplay between tumor cells and immune cells (Yang et al., 2022). Additionally, *LCK*, a crucial component of T cell receptor signaling pathways, may influence T cell activation and anti-tumor immune response (Wu et al., 2021). Furthermore, *BRCA1*, beyond its established role in DNA repair, may affect immune surveillance mechanisms and the tumor’s ability to evade immune recognition through its involvement in maintaining genomic stability (Yoshida and Miki, 2004). Further investigation into the mechanisms by which specific genes within the 14-gene signature interact with the immune microenvironment could unveil novel therapeutic targets, enabling the optimization of immunotherapy strategies. For these high-risk patients, potential treatment modifications could include intensified neoadjuvant regimens with additional cycles, closer monitoring during treatment, and more frequent surveillance schedules during follow-up.

Despite highlighting the significant value of the 14-gene molecular assay in evaluating the efficacy of nICT in NSCLC patients, this study has notable limitations. First, as a single-center retrospective study with a relatively small sample size, the potential for selection bias cannot be overlooked. Our current study represents an important initial step by demonstrating the signature’s predictive capability in a real-world clinical cohort, but external validation in independent cohorts across diverse patient populations and different institutions is essential for clinical implementation. Only through rigorous external validation can we ensure the signature’s robustness and reliability across different clinical settings before considering its integration into routine clinical practice. Second, the 14-gene molecular assay was conducted exclusively on post-surgical tumor tissue samples, with no analysis of pre-neoadjuvant treatment biopsy specimens. This limitation hinders the dynamic assessment of changes in gene expression before and after treatment and their potential impact on therapeutic efficacy. Third, given that the original 14-gene assay was primarily validated in early-stage NSCLC patients, its direct application to the neoadjuvant treatment context may benefit from model recalibration to optimize predictive performance. Nevertheless, we believe the 14-gene signature’s predictive value remains relatively stable across different treatment regimens because it captures intrinsic tumor biological characteristics that are more deterministic of treatment response than treatment variations themselves. Furthermore, the study did not comprehensively investigate the mechanisms of interaction between specific genes within the 14-gene signature and the immune microenvironment. Future research should prioritize larger-scale, multi-center prospective studies, integrating advanced technologies such as single-cell sequencing and spatial transcriptomics. These efforts would allow for a more in-depth exploration of the relationship between the 14-gene signature and the tumor immune microenvironment, ultimately providing more precise and tailored guidance for the individualized treatment of NSCLC patients.

## 5 Conclusion

In conclusion, this study highlights the significant value of the 14-gene molecular assay in assessing the efficacy of nICT for NSCLC. Additionally, the 14-gene risk stratification demonstrates a clear association with TLS and TIL within the immune microenvironment after nICT. The 14-gene molecular signature holds promise as a valuable tool for personalized medicine, enabling pre-treatment identification of high-risk patients, guiding individualized treatment decisions and follow-up strategies, and providing clinicians with molecular-level prognostic information that complements conventional TNM staging. Future research should focus on conducting large-scale multicenter prospective validation studies, exploring the molecular mechanisms underlying 14-gene interactions with the tumor immune microenvironment, integrating multidimensional biomarkers to construct more precise prognostic models, and establishing standardized testing procedures and clinical application guidelines to facilitate clinical translation. These findings collectively indicate that the 14-gene molecular signature represents a promising advancement toward optimizing efficacy evaluations of neoadjuvant therapy in NSCLC, potentially facilitating the development and implementation of patient-specific therapeutic algorithms and precision treatment stratification approaches in clinical practice.

## Data Availability

The original contributions presented in the study are included in the article, further inquiries can be directed to the corresponding authors.
